# Terahertz Technology: A Boon to Tablet Analysis

**DOI:** 10.4103/0250-474X.56014

**Published:** 2009

**Authors:** M. P. Wagh, Y. H. Sonawane, O. U. Joshi

**Affiliations:** NDMVPS's College of Pharmacy, Department of Pharmaceutics, Shivajinagar, Gangapur Road, Nashik-422 002, India

**Keywords:** Coating uniformity, film coating, process analytical technology (PAT), Terahertz pulsed imaging (TPI)

## Abstract

The terahertz gap has a frequency ranges from ∼0.3 THz to ∼10 THz in the electromagnetic spectrum which is in between microwave and infrared. The terahertz radiations are invisible to naked eye. In comparison with x-ray they are intrinsically safe, non-destructive and non-invasive. Terahertz spectroscopy enables 3D imaging of structures and materials, and the measurement of the unique spectral fingerprints of chemical and physical forms. Terahertz radiations are produced by a dendrimer based high power terahertz source and spectroscopy technologies. It resolves many of the questions left unanswered by complementary techniques, such as optical imaging, Raman and infrared spectra. In the pharmaceutical industries it enables nondestructive, internal, chemical analysis of tablets, capsules, and other dosage forms. Tablet coatings are a major factor in drug bioavailability. Therefore tablet coatings integrity and uniformity are of crucial importance to quality. Terahertz imaging gives an unparalleled certainty about the integrity of tablet coatings and the matrix performance of tablet cores. This article demonstrates the potential of terahertz pulse imaging for the analysis of tablet coating thickness by illustrating the technique on tablets.

Despite the ongoing development of more sophisticated solid drug delivery systems, tablets are still by far the most prevalent solid dosage form. Not all active pharmaceutical ingredients (API) inherit favorable physicalchemical characteristics for production, storage and administration, thus requiring dosage form modifications such as coating. Coating can improve taste, aesthetic appearance or mask odour. In addition to this, tablets are often coated with a therapeutic purpose. For example, enteric coating is used to protect the API against degradation in the stomach and sustained-release coating is used to obtain a desirable API absorption rate, and hence an optimum plasma-release profile[1]. The quality of coating properties such as, layer thickness, uniformity and reproducibility of the tablet coating have direct implications on product performance. Lack of quality resulting in dose failures such as dose dumping may precipitate legal and commercial consequences for the manufacturer. It is hence of great interest for these coating properties to be monitored and controlled during the production process. Routinely in the pharmaceutical industry, tablet coating is controlled by employing calculations on tablet weight-gain during the coating processes with respect to the amount of coating solution applied. However, weight gain determination does not give information on coating uniformity[2]. Other techniques such as scanning electron microscopy, conventional optical microscopy, and laser induced breakdown spectroscopy may be used but are destructive techniques and consequently difficult to apply in in-process quality control[3]. New developments in scanning electron microscopy do allow the non-destructive characterization of film coatings under environmental conditions, and hence can provide information on certain film coating properties. However, using this technique is not possible to acquire information on the coating layer thickness without sample destruction. Near infrared (NIR) and Raman analysis have also been employed to analyze coating thickness and variability, however these techniques are often restricted by their need for chemometric models, and provide information biased to the surface of the coating. Moreover, only construction of two dimensional chemical images is within the capability of these techniques[3–7]. Magnetic resonance imaging (MRI) can be used to monitor the diffusion of dissolution medium into coating structures and the tablet matrix[8]. Its ability to image liquids makes MRI an excellent tool to perform quantitative *in situ* studies of drug release, disintegration and change in pore size during dissolution[9]. Nevertheless due to the short relaxation times of typical pharmaceutical solids, in MRI the signal is only acquired from the liquid thus information of the coating structure or all other solid is obtained indirectly. To date, terahertz radiation has been established for pharmaceutical applications when used as a spectroscopic technique in polymorph identification and quantification[10–12], phase transition monitoring[13–14] and hydrates recognition[15]. Since various techniques for terahertz pulsed imaging (TPI) were pioneered relatively recently[16–17], the application of terahertz pulsed imaging in the pharmaceutical sciences was only explored in chemical mapping[18,19] and tablet coat imaging[20] thus far. This initial work showed the usefulness of this technique for coating thickness analysis and demonstrated the non-destructive nature of TPI due to its ability to penetrate through most pharmaceutical excipients, yet at the same time resolving internal structure by detecting subtle changes in the refractive index. With the implementation of a six-axis robotic system specifically designed for the fully automated analysis of pharmaceutical solid dosage forms, imaging in the terahertz range (0.3–3.0 THz or 10–100 cm^−1^) is now readily available as illustrated by Zeitler *et al.*[21].

## Terahertz Technology:

Terahertz radiations have a few remarkable properties. Many common materials and living tissues are semitransparent and have ‘terahertz fingerprints’, permitting them to be imaged, identified and analyzed. Due to non-ionizing properties of terahertz radiations are safe for screening application. These unique properties of radiations are now exploited due to availability of commercial sources of terahertz radiations.

**Fig. 1:**
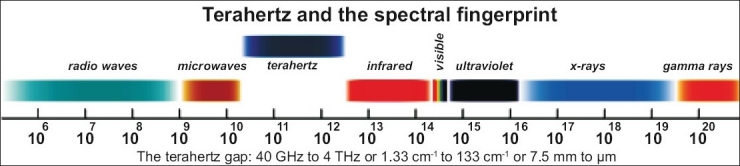
electromagnetic spectrum of terahertz radiation (courtesy of SURA).

Terahertz pulsed imaging (TPI) is a nondestructive analysis tool which utilizes the latest technology to generate and detect ultra short pulses of terahertz energy (0.06-3.6 THz, 2-120 cm^−1^) as shown in fig. 1. The technique operates much the same way as ultrasound or radar is used to accurately locate embedded or distant objects. Like these techniques, the sample itself is unaffected by the measurement. Three dimensional (3D) TPI combines terahertz spectroscopic measurements with refractive index measurements of chemical/physical interfaces to produce 3D chemical maps. Three hundred and sixty degrees-3D images of tablets were collected on TeraView's TPI image 2000 System. This terahertz system is equipped with a robotic arm which moves the tablet in up to five axes for complete 360°-3D imaging of tablets. Terahertz pulses incident on a tablet surface penetrate through the different coating layers. At each interface or change in refractive index, a portion of the terahertz pulse is reflected back to the detector. The amplitude of reflected terahertz radiation is recorded as a function of time. Most pharmaceutical coating materials are semi-transparent in the terahertz spectral region and, as the macroscopic structure within the coating is much less than the wavelength of the radiation, contributions due to light-scattering are not significant. Three dimensional images consist of the horizontal and vertical spatial dimensions combined with the time delay (depth profile) of the tablet[21].

## Pharmaceutical Industry; Tablet integrity and performance:

Terahertz image can be optimized for performing 3D analysis on tablets. It can enable determination of coating integrity and thickness, detect and identify localized chemical or physical structure such as cracks or chemical agglomeration within a core and to interrogate embedded layers (such as an interface between two layers) for delamination and integrity.

Terahertz measurements may well become the primary method for the nondestructive determination of coating thickness, requiring little or no calibration for most coatings and substrates. It can reveal the thickness, uniformity, distribution and coverage of simple and complex coating. Terahertz image can also detect embedded layers and localized chemical or physical structural features in the cores of intact tablets to confirm 3D morphology and blend uniformity.

## Why coating analysis?

Coating has a wide variety of functions. The most important function of coating is to regulate the controlled release of active ingredients in the body. Coating not only contributes to the bioavailability of a particular drug or combination of drugs during certain times and locations but also coating can protect the stomach from high concentrations of active ingredients, improve tablet visual appeal and extend shelf life by protecting the ingredients from degradation by moisture and oxygen. In relation to tablet coating the process analytical technology (PAT) initiative is intended to improve consistency and predictability of tablet action by improving quality and uniformity of tablet coatings. Issues with coating can arise from problems with the coating materials or flaws in the coating pan or spray process. If a coating is non-uniform or has surface defects then the desired dose delivery and bioavailability can be compromised. From this standpoint it is important to characterize tablet coating uniformity, both within a single tablet and across an entire batch to develop an understanding of the functional analysis of the final product[21].

## Coating analysis:

Several analytical and imaging techniques are being used to understand the critical processes involved in tablet coating but none of them is ideal to fully characterize the layers. Some of the techniques that provide useful information are atomic force microscopy, confocal laser microscopy, X-ray photoelectron spectroscopy, electron paramagnetic resonance, fourier transform infrared spectroscopy, and laser-induced breakdown spectroscopy (LIBS)[22] and scanning thermal microscopy. However all these methods are either destructive to the tablet or cannot be readily implemented for rapid on-line measurement.

## Terahertz pulsed imaging (TPI):

TPI is a completely non-invasive and non-destructive pharmaceutical analysis tool using extremely low power, ultra short pulses of electromagnetic radiation at lower frequencies than infrared (1 THz= 1012 Hz). Terahertz spectroscopy has already proved useful to distinguish between different polymorph forms of the drug[23]. TPI is a next step of this whereby THz pulses are used to image object of interest. THz pulses are generated by illuminating photoconductive semi-conductors with pulsed near-infrared laser radiation and detected coherently[24]. Tablet coatings are semi-transparent to THz frequencies and do no scatter them significantly. THz pulses incident on a tablet surface penetrate through the different coating layers. At each interface a portion of THz radiations is reflected back to the detector. The amplitude of reflected THz radiation is recorded as a function of time. In this technique the sample itself is completely unaffected by the measurement. Coating thickness uniformity is established simply from the transit time of the pulse to each interface. With knowledge of the refractive index of coating material the actual thickness can be determined to a depth resolution of about 20 microns. The spot size of the THz pulse, and therefore lateral resolution, is about 250 microns.

## Advantages of TPI over other techniques:

TPI has many advantages over the few existing techniques for investigating coatings. It is a completely non-invasive investigative tool that has similar depth resolution and higher lateral resolution than LIBS. In addition, because it is non-destructive, tablet can be re-examined at later times to monitor stability or used for further functional studies with prior knowledge of the coating uniformity. Any number of locations in tablet can be probed by TPI, and the measurement can be extended to a 3D image if desired.

## EVIDENCES

### Analysis of coating layers in ibuprofen tablet:

In this application scientists demonstrated the potential of TPI for the analysis of tablet coating thickness a leading brand of ibuprofen tablets and a generic version. The TPI system was used to probe both branded and generic ibuprofen tablets. THz pulses were directed onto the tablet and penetrated through each of the layers. The signal reflected by the coating interfaces was measured as a function of time. Coating depth can be derived from the time domain measurement by calibrating for the refractive indices of the coating materials[25]. With TPI it is possible to analyze any region on the tablet, and in this case the THz beam was raster scanned across a square section of the tablet 1 mm by 1 mm. The THz time domain data can be analyzed and displayed in a number of formats to characterize coating uniformity. In the simplest format, a typical trace from a single point in the tablet is plotted. The uniformity of each layer can be determined from the time at which the reflected pulses are observed. In agreement with microscope photographs these waveforms clearly show that the brand tablet has several coating layers, and the overall thickness is greater than the generic one. After calibration of the time axis using refractive index estimation the total thickness of coatings are determined as 440 microns for the brand tablet and 305 micron for the generic one.

The uniformity and consistency of the ibuprofen tablet coating is better conveyed by an image of the depth profile, analogous to the typical β-scan ultrasound format. These images clearly show the difference in structure and thickness of the two coatings. The variations observed in the microscope image for the brand tablet, in particular the light central band within the darkest layer can be apparently seen. This would be missed by a technique that made measurements at selected single point locations apparent in the microscope photograph. The analysis can be extended and the tablet coatings mapped in 3D, using surfaces to indicate the positions of the layers. In the case of TPI, we mount the THz measurement device on a computer controlled robot arm to position the so that the beam is normal to the surface at every measurement location. This results in an accurate depth profile for all geometries.

## OTHER TETRAHERTZ APPLICATIONS

### Molecular structure:

The sensitivity and specificity of Terahertz spectroscopy to both intermolecular and intramolecular vibrations in different chemical species enable investigation of the crystalline state of drugs e.g. polymorphism. The use of pulsed terahertz imaging in proteomics and drug discovery determines protein 3D structure, folding and characterization. It is also very sensitive to DNA hybridization and other interactions. Terahertz spectroscopy provides rapid identification of the different crystalline forms of drug molecules, the polymorphs, which can exhibit different solubilities, stabilities and bioavailability and hence are an important factor in the therapeutic efficacy of a drug. Detecting and identifying the different polymorphs and understanding the mechanism and dynamics of polymorphic inter-conversion, is an important milestone in selecting the optimum form for further development and manufacture[26]. It is possible not only to detect the differences between pure specimens of the polymorphs but terahertz spectroscopy can distinguish between specific polymorphic forms in tablet formulation. Terahertz spectroscopy can also differentiate between different hydrate forms. Lactose which is one the most commonly used excipients in the pharmaceutical industry has at least three different hydrates namely α-monohydrate, α-anhydrate and β-anhydrate form. These three hydrate forms exhibit terahertz spectra that can be used for both quantitative and qualitative analysis. Terahertz region provides unique sensitivity to lattice structure enabling qualitative and qualitative analysis of crystalline and amorphous materials as well.

### Time resolved THz spectroscopy of protein folding:

Proteins fold catalyze reactions, and transducer signals via binding to other biomolecules. These processes are driven by motions with characteristics time scales ranging from femtoseconds (fs) to milliseconds (ms). The characteristic modes from which such motions collectively emerge often cause large amplitude deformations of all or part of the protein. Temperature tuning reveals when certain modes are frozen out, while the Terahertz spectroscopy can cover fast relaxation kinetics on fs time scale during which a protein rearranges its overall structure.

### In dermatology:

The cosmetic appearance of skin is directly linked to the outermost layer, the stratum corneum. The water content of the stratum corneum influences its permeability and elasticity. Most skin-care products such as moisturizers act to increase the retained water content of this layer of the skin to enhance its appearance. Quantitative characterization of the hydration level of the stratum corneum is thus of crucial importance to the cosmetic industry in order to characterize and compare the effectiveness of their products.

### Oral healthcare:

Dental carries or tooth decay is one of the most common human disorders. Carries proceed by the creation of a subsurface lesion in the enamel. The lesion may extend to the next tissue in the layer in teeth, the dentine, without macroscopically visible breakdown or even microcavity formation at the tooth surface. The absence of visual features on the tooth surface makes early detection of tooth decay difficult. X-rays which is one of the accepted methods used to detect decay, only reveals the problem at a relatively late stage, when drilling and filling is the only method available to halt the decay[25]. If decay can be detected early enough it is possible to reverse the process without the need for drilling by the use of either fissure sealing or remineralisation. Terahertz imaging can distinguish between the different types of tissue in a human tooth; detect carries at an early stage in the enamel layers of human teeth and monitor early erosion of the enamel at the surface of the tooth.

### Oncology:

It is estimated that more than 85% of all cancers originate in the epithelium. Excision biopsy to remove tissue from the body and examining it under a microscope is the gold standard for cancer diagnosis. Terahertz technology has the potential to greatly improve conventional biopsy and associated surgery by more precisely identifying the areas to be excised thereby reducing the number of procedures and facilitating earlier and more accurate diagnosis. As the technology develops, it may be possible to perform biopsies using live terahertz imaging of affected area, making possible point of care optical biopsy.

### Chemical and bioagent detection through spectroscopy:

Terahertz imaging could critical as a means of screening for explosives or other prohibited or dangerous chemicals. Terahertz rays can provide spectroscopic information on the object in question as they have capabilities of structural imaging modality. From these details of the object's chemical composition may be determined. This has important advantage in the fight against terrorism and detection of suspicious or prohibited substances.

### Screening:

The ability of terahertz technology to see through many opaque things such as clothing box, shoe has led to successful proof of experiments at standoff distances for potential applications in building security, airports, and defense.

## MINOR APPLICATIONS OF TETRAHERTZ

There are other applications of terahertz spectrometry and imaging. Some important ones to be mentioned are food industry process control (e.g. moisture detection), earth remote sensing, environmental sensing (pollution detection), plasma diagnostics, seeing through sand storms, active and passive imaging through dust, smoke and fog.[27]; all weather active and passive seekers; secure communications; spectrographic sensing of explosives, gases and biologicals. Terahertz imaging can be useful in high rate and secure data transfer, flame analysis (rocket or jet engine burn optimization), homeland security–concealed weapon identification, detection of suicide bombers, biological threat detection, detection of voids in the space shuttle foam and in other structural materials, passenger screening, hidden weapon detection, contraband detection.

### Regulatory relevance of pharmaceutical characterization:

Pharmaceutical characterization is one critical aspect for a successful product and process development program, as it provides important physical, chemical, structural and property-related information of pharmaceutical components in formulated products. For both drug substances and drug products, there are pertinent regulations described in CFR 21 §314.50(d)(1) Chemistry, Manufacturing, and Controls section to assure the identity, strength, quality, purity, and bioavailability of the drug product. However for the most part, more characterization emphasis has traditionally been put on API while less effort has focused on excipients, due perhaps to inadequate appreciation of the various roles excipients play in formulation and process. The gap between the CFR requirements and current industry practices in some cases may present potential risk to the public health in the event of introducing new excipient to a formulation during development[26] and unexpected appearance or disappearance of a crystalline form[27]. The scientific significance and regulatory relevance of pharmaceutical polymorphism characterization and pharmaceutical crystallization process control using PAT have been discussed in detail in the recent literature[22,23,28]. Advanced process analyzers and spectroscopic techniques have been used for pharmaceutical applications[24,29] for probing molecular vibrational modes in the near- and mid-infrared regions of the electromagnetic spectrum for many years. However, studies using far-infrared or Terahertz (FIR or THz, 25-300 micrometer wavelength or 3-500 cm^−1^) spectroscopic techniques had been limited due to the difficulty in accessing this low frequency range, especially below 50 cm^−1^.

## CONCLUSION

In this review, measurement process conditions and PAT considerations of using THz spectroscopy to characterize pharmaceutical materials and tablets were explored with the goal of evaluating THz spectroscopy as an emerging technology for potential pharmaceutical PAT application. On the basis of the study carried out by Wu *et al.* which revealed that three factors including component loading, component chemistry, and disk drying time have significant impacts on the raw THz transmission spectra of crystalline materials and amorphous materials.

Terahertz spectroscopy is of prime importance for tablet coating technology. At present 3D TPI™ provides the ability to nondestructively and rapidly analyze the coating layer thickness and quality of coated pharmaceutical tablets. Most important advantage of Terahertz technology is that it is nondestructive method of analysis. Tablets can be re-examined at later times to monitor coating stability or used for further functional studies with prior knowledge of the coating uniformity.
